# Turbulent Intensity of Blood Flow in the Healthy Aorta Increases With Dobutamine Stress and is Related to Cardiac Output

**DOI:** 10.3389/fphys.2022.869701

**Published:** 2022-05-25

**Authors:** Jonathan Sundin, Mariana Bustamante, Tino Ebbers, Petter Dyverfeldt, Carl-Johan Carlhäll

**Affiliations:** ^1^ Unit of Cardiovascular Sciences, Department of Health, Medicine and Caring Sciences, Linköping University, Linköping, Sweden; ^2^ Center for Medical Image Science and Visualization, Linköping, Sweden; ^3^ Department of Clinical Physiology in Linköping, Department of Health, Medicine and Caring Sciences, Linköping University, Linköping, Sweden

**Keywords:** 4D flow MRI, aortic blood flow, cardiovascular magnetic resonance, dobutamine stress, turbulent blood flow

## Abstract

**Introduction:** The blood flow in the normal cardiovascular system is predominately laminar but operates close to the threshold to turbulence. Morphological distortions such as vascular and valvular stenosis can cause transition into turbulent blood flow, which in turn may cause damage to tissues in the cardiovascular system. A growing number of studies have used magnetic resonance imaging (MRI) to estimate the extent and degree of turbulent flow in different cardiovascular diseases. However, the way in which heart rate and inotropy affect turbulent flow has not been investigated. In this study we hypothesized that dobutamine stress would result in higher turbulence intensity in the healthy thoracic aorta.

**Method:** 4D flow MRI data were acquired in twelve healthy subjects at rest and with dobutamine, which was infused until the heart rate increased by 60% when compared to rest. A semi-automatic segmentation method was used to segment the thoracic aorta in the 4D flow MR images. Subsequently, flow velocity and several turbulent kinetic energy (TKE) parameters were calculated in the ascending aorta, aortic arch, descending aorta and whole thoracic aorta.

**Results:** With dobutamine infusion there was an increase in heart rate (66 ± 9 vs. 108 ± 13 bpm, *p* < 0.001) and stroke volume (88 ± 13 vs. 102 ± 25 ml, *p* < 0.01). Additionally, there was an increase in Peak Average velocity (0.7 ± 0.1 vs. 1.2 ± 0.2 m/s, *p* < 0.001, Peak Max velocity (1.3 ± 0.1 vs. 2.0 ± 0.2 m/s, *p* < 0.001), Peak Total TKE (2.9 ± 0.7 vs. 8.0 ± 2.2 mJ, *p* < 0.001), Peak Median TKE (36 ± 7 vs. 93 ± 24 J/m3, *p* = 0.002) and Peak Max TKE (176 ± 33 vs. 334 ± 69 J/m3, *p* < 0.001). The relation between cardiac output and Peak Total TKE in the whole thoracic aorta was very strong (R^2^ = 0.90, *p* < 0.001).

**Conclusion:** TKE of blood flow in the healthy thoracic aorta increases with dobutamine stress and is strongly related to cardiac output. Quantification of such turbulence intensity parameters with cardiac stress may serve as a risk assessment of aortic disease development.

## Introduction

Normal cardiovascular blood flow is predominately laminar. However, cardiovascular diseases often affect blood flow patterns in the major vessels and cardiac chambers, and can cause transitionally turbulent flow. Specifically, diseases such as aortic valve stenosis and aortic coarctation are associated with murmur-producing turbulent flow in the aorta (Sabbah and Stein 1976; Murgo 1998; Rao 2005). Turbulent flow may also be present in healthy individuals but to a smaller degree and usually not enough to produce a heart murmur (Sabbah and Stein 1976).

Turbulent flow is not only a consequence of pathologies in the heart and aorta. It can also affect and damage tissues in the cardiovascular system. Blood flow affects endothelial cells which play an important role in e.g., atherogenic and hemostatic processes. The patterns of shear stress seen in steady laminar flows cause endothelial cells to align in the direction of the flow and also stimulates the endothelial cells to express athero-protective proteins. In turbulent flow, the shear stress on the vessel wall is discontinuous, the cells fail to align in the flow direction, and the athero-protective expression of proteins changes to an atherogenic expression (Davies et al., 1986; Davies 1995; Chiu and Chien 2011). Moreover, turbulent flow has also been associated with damage to blood cells and loss of function of proteins important to hemostasis (Sutera 1977; Hollestelle et al., 2011).

Three-dimensional, time-resolved, phase-contrast magnetic resonance imaging (4D flow MRI) is a technique that employs motion encoding in all three spatial directions over time to enable detailed characterisation of multidimensional blood flow within the cardiovascular system throughout the cardiac cycle. One promising 4D flow MRI based parameter is the turbulent kinetic energy (TKE), which is the kinetic energy of turbulent velocity fluctuations and can be computed from the intravoxel velocity standard deviation in three spatial directions (Dyverfeldt et al., 2006, 2015). TKE in the aorta and heart has been investigated in patients with aortic valve stenosis (Dyverfeldt et al., 2008; Binter et al., 2013; Dyverfeldt et al., 2013; Binter et al., 2017), aortic coarctation (Dyverfeldt et al., 2008; Arzani et al., 2012; Lantz et al., 2013), hypertrophic cardiomyopathy (Iwata et al., 2020) and aorta of normal individuals (Ha et al., 2018).

Cardiac MRI stress test with dobutamine as a pharmacological agent is well established to expose abnormalities in cardiovascular function (Monmeneu Menadas et al., 2016). However, studies on cardiac stress test with 4D flow MRI are sparse. In this study, we sought to investigate how heart rate and inotropy affect TKE parameters in the healthy thoracic aorta under cardiac stress by infusing dobutamine. We hypothesized that dobutamine stress would result in higher turbulence intensity.

## Methods

### Study Population

Twelve healthy subjects (eight women) with a normal physical examination, no history of cardiovascular disease or medication for cardiovascular disease and with an ordinary level of physical activity were included in the study. The exclusion criteria were: abnormal cardiac dimensions, abnormal cardiac wall motion or severe aortic valve stenosis based on balanced steady-state free precession (bSSFP) CMR data at rest. The study was approved by the Regional Ethical Review Board in Linköping and complies with the Declaration of Helsinki. All subjects provided written informed consent prior to participation in the study.

### Study Protocol

**FIGURE 1:**
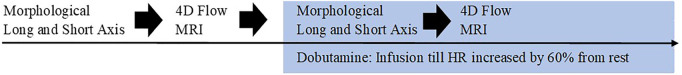
MRI protocol. Morphological images and 4D flow data collected at rest and with dobutamine infusion; HR, Heart Rate.

The subjects completed a CMR examination consisting of two parts. The first part at rest with no intervention followed by the second part during infusion of dobutamine (Figure 1). Dobutamine was administered intravenously with a starting dose between 5 and 10 μg/kg/min. The target heart rate was 60% higher than the subject’s heart rate at rest and the dose was increased or decreased approximately every 2 minutes to reach and maintain the target heart rate. Dobutamine administration was maintained until the CMR data in the second part were acquired. Heart rate was monitored continuously throughout the examination, blood pressure was measured at rest and approximately every second minute during dobutamine administration, using a cuff sphygmomanometer. Criteria for interrupting the dobutamine infusion were any discomfort due to dobutamine or achievement of maximal systolic blood pressure (220 mmHg).

### CMR Data Acquisition

4D flow MRI data of the heart and aorta, as well as cine bSSFP morphological images of the left ventricle were acquired using a 3T Phillips Ingenia scanner (Phillips Healthcare, Best, the Netherlands). The 4D flow data were acquired using a free breathing navigator-gated gradient-echo pulse-sequence with interleaved three-directional flow-encoding and retrospective vector cardiogram controlled cardiac gating (Eriksson et al., 2010). Velocity encoding (VENC) 140 cm/s, repetition time 5.2 ms, flip angle 5°, echo time 3.0 ms, k-space segmentation factor 2, elliptical k-space acquisition, parallel imaging (SENSE) speed up factors 1.6 (RL direction), and 3 (AP direction), weighted navigator gating with 4 mm in the inner 25% of k-space and 7 mm in the 75% outer part of k-space was used (Dyverfeldt and Ebbers 2017). The temporal resolution was 41.6 ms and the spatial resolution was 2.8 × 2.8 × 2.8 mm^3^. Scan time was approximately 7–8 min including navigator efficiency, for a nominal heart rate of 60 bpm.

The bSSFP images were acquired during end-expiratory breath holds and included three- and four-chamber long axis and a stack of short-axis images at rest. At dobutamine infusion, three-chamber long axis and a stack of short-axis images were acquired. The bSSFP images were reconstructed into 30 timeframes with a resolution for the short-axis images of 0.9 × 0.9 mm^2^ and for the long-axis images 0.83 × 0.83 mm^2^. Scan parameters included: slice thickness 8 mm, echo time 1.4 ms, repetition time 2.8 ms, flip angle 45°.

### Data Analysis

**FIGURE 2:**
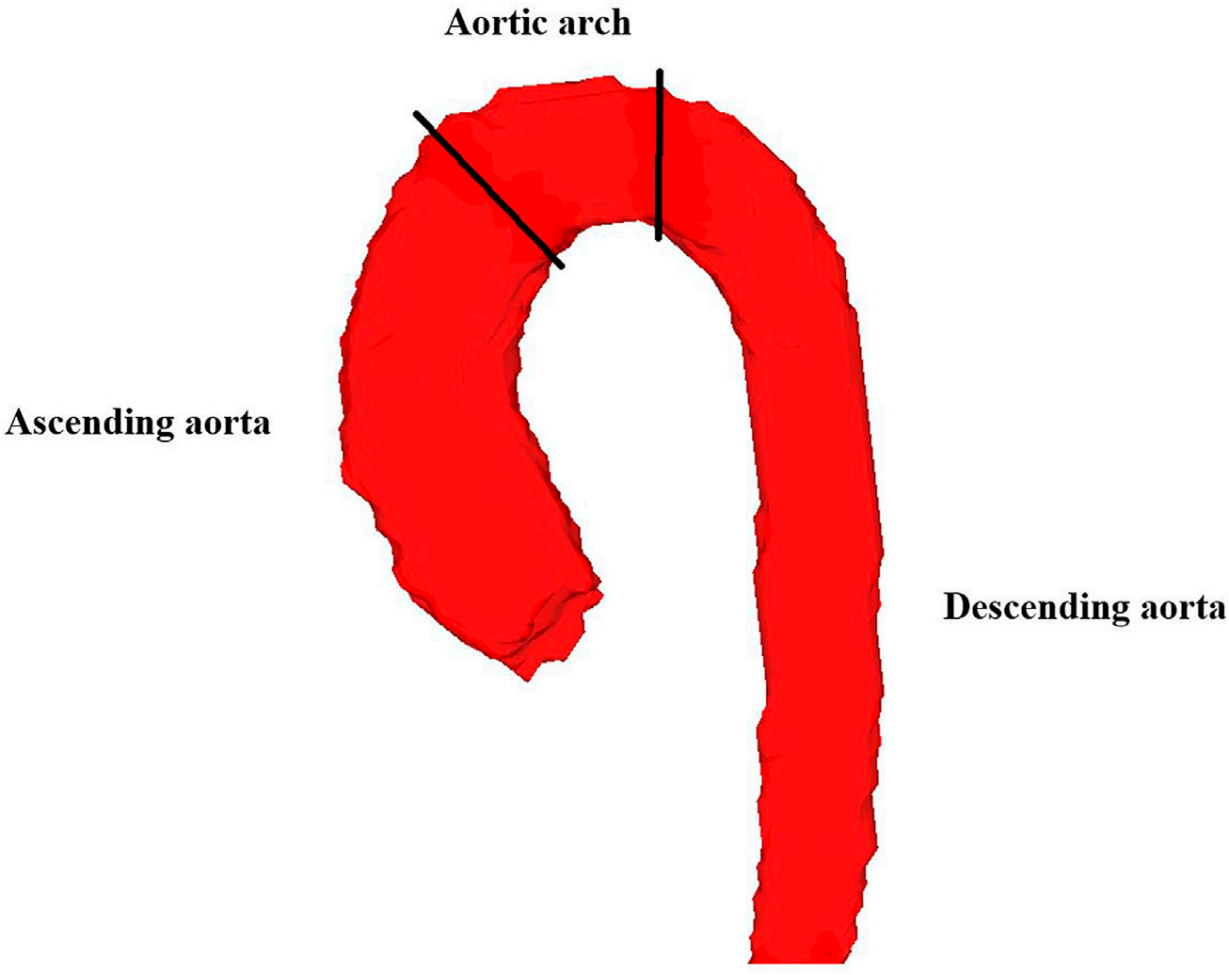
Segmentation of the thoracic aorta in one of the healthy subjects. The whole thoracic aorta was divided into three regional volumes; ascending aorta, aortic arch and descending aorta. The boundaries between these regional volumes are illustrated with black lines.

TKE was obtained as described previously (Dyverfeldt et al., 2006, 2008). Briefly, MR turbulence mapping uses an MR signal model to relate signal loss effects to the intravoxel velocity standard deviation. TKE is calculated from the intravoxel velocity standard deviation in three directions. Phase unwrapping was performed using automatic temporal (Wigstrom et al., 1999) and 4D Laplacian (Loecher et al., 2016) unwrapping, and was complemented by manual unwrapping when these algorithms were not able to unwrap all voxels. Segmentations over the whole cardiac cycle were generated for the 4D flow data using an automated multi-atlas segmentation tool (Bustamante et al., 2018). This method uses 4D Phase-contrast Magnetic Resonance CardioAngiographies (4D PC-MRCA) generated from each 4D Flow MR acquisition (Bustamante et al., 2017). Eight manually segmented atlases are transferred to all 4D PC-MRCA datasets at end-diastole and end-systole using a non-rigid registration. A fusion algorithm combines these different atlas segmentations into a final segmentation, after which a fully time-resolved segmentation of the 4D Flow MRI data is obtained by registration between the remaining time points. Validation of the segmentation method in the aorta has been performed in both patients and healthy volunteers through flow analyses of different locations in the aorta and comparison of systemic volume flows (Qs) with pulmonary flows (Qp) (Bustamante et al., 2015, 2018). The aortic segmentations generated with the tool were inspected in 2D and 3D and manually corrected when necessary with the software ITK-snap (Yushkevich et al., 2006). The whole thoracic aorta (TAo) was divided into three regional volumes: I) Ascending aorta (AAo) from the aortic valve reaching to the brachiocephalic trunk, II) Aortic arch (AoA) from the brachiocephalic trunk to the left common carotid artery, and III) Descending aorta (DAo) from the left common carotid artery to where the aorta exits the thorax, at the level of the diaphragm (Figure 2). An inhouse developed tool for assessment of hemodynamic parameters was utilized.

**FIGURE 3:**
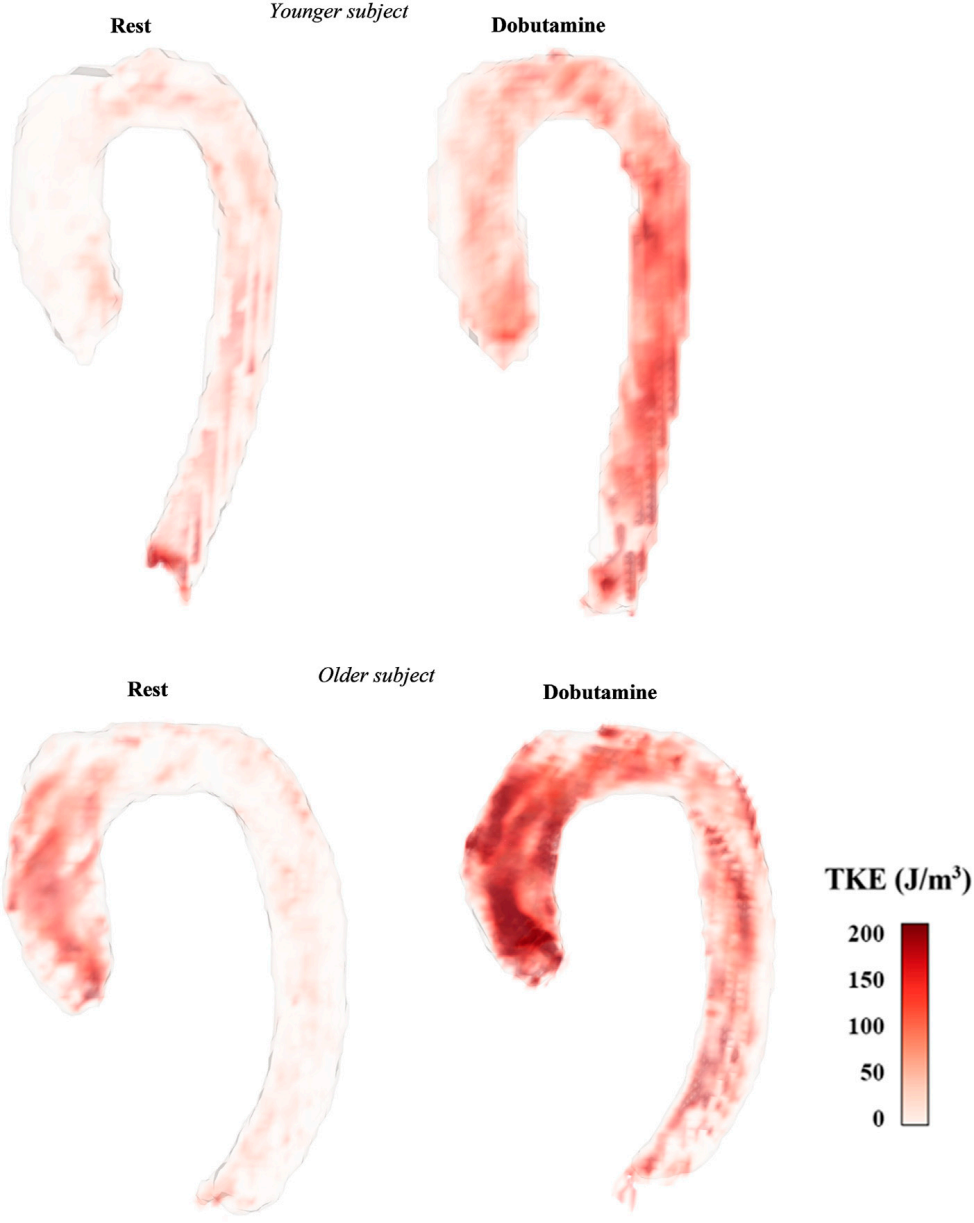
Map of TKE at the timeframe of TAo peak TKE_total_ in two of the healthy subjects with different TKE patterns and different age (younger = 20 years old, older = 56 years old).

Several hemodynamic parameters were computed in the four segmented regions for every timeframe: average speed (V_avg_), maximum speed (V_max_), total TKE (TKE_total_), maximum TKE (TKE_max_) and median TKE (TKE_med_). The parameters’ peak values in the cardiac cycle were identified. TKE_total_ was computed as the volume integral of TKE in each region. TKE_max_ was defined as the maximum TKE in any voxel in a given region. Finally, TKE_med_ was defined as the median TKE in a given region. TKE_total_, TKE_max_, and TKE_median_ at the time point with the highest TKE_total_, TKE_max_, and TKE_median_ were termed peak TKE_total_, peak TKE_max_, and peak TKE_median_, respectively (Figure 3). Median filtering with a 3 × 3 × 3 kernel was used to reduce the impact of noise on the assessment of peak TKE_max_.

The morphological bSSFP images were segmented to obtain left ventricular end-diastolic volume (LVEDV) and left ventricular end-systolic volume (LVESV) using the freely available software Segment version 1.9 (Medviso, Lund, Sweden) (Heiberg et al., 2010).

Inter- and intraobserver variability analyses were performed for velocity and TKE parameters at dobutamine stress, with intra-class correlation coefficient (ICC) estimates based on mean-rating, absolute-agreement and 2-way mixed-effects model. Reader 1 and reader 2 had both approximately 5 years of experience in cardiovascular imaging. There was a washout period of more than 4 weeks for the intraobserver variability analysis (reader 1).

### Statistical Evaluation

The Shapiro-Wilks test was used to assess if the data was normally distributed. For the normally distributed data, t-tests for paired samples were performed. In the cases when the data was not normally distributed, a Wilcoxon signed-rank test was performed. For intergroup comparisons, a one-way analysis of variance (ANOVA) with a Tukey post-hoc test was used for normally distributed data. Kruskal–Wallis with Dunn’s post-hoc test was performed for intergroup comparisons when the data was not normally distributed. Linear regression was used to analyse how cardiac output, stroke volume and heart rate were related to TKE. Results are given as group mean ± SD and a *p*-value < 0.05 was considered significant. The software used for all statistical analyses was SPSS v. 27.0 (IBM, Armonk, NY, United States).

## Results

No complications were observed due to the infusion of dobutamine, and all examinations were completed. The peak dose of infused dobutamine for the study cohort was 22 ± 6 μg/kg/min (range 15–30 μg/kg/min). Demographic data and clinical parameters for the twelve subjects are shown in Table 1. With the infusion of dobutamine, heart rate and systolic blood pressure increased while diastolic blood pressure had no significant change. The left ventricular volumes decreased with the infusion; however, LVEDV had a lesser reduction than LVESV, which resulted in an increase in stroke volume. Ejection fraction and cardiac output both increased. The volumes of the thoracic aorta were not different between rest and stress at the time point of Peak TKE_total_ (Table 2).

**TABLE 1:** Demographic and clinical parameters.

	Rest	Dobutamine	*p*-Value
Age (years)	33 ± 13		
Gender (f/m)	8/4		
Height (cm)	172 ± 8		
Weight (kg)	68 ± 8		
HR (bpm)	66 ± 9	108 ± 13	<0.001
BP systolic (mmHG)	118 ± 13	135 ± 15	0.021
BP diastolic (mmHG)	68 ± 9	63 ± 8	0.251
LVEDV (ml)	153 ± 28	139 ± 35	0.004
LVESV (ml)	65 ± 17	36 ± 13	<0.001
LVEF (%)	58 ± 5	74 ± 5	<0.001
LVSV (ml)	88 ± 13	102 ± 25	0.010
CO (L/min)	5.8 ± 1.0	11.0 ± 2.4	<0.001

HR, heart rate; BP, blood pressure; LV, left ventricle; EDV, end-diastolic volume; ESV, end-systolic volume; EF, ejection fraction; SV, stroke volume; CO, cardiac output.

**TABLE 2:** Volumes for all regions of the thoracic aorta at Peak TKE_total_.

Volume (ml)	Rest	Dobutamine	*p*-Value
Ascending aorta	35.4 ± 9.3	35.7 ± 9.7	= 0.538
Aortic arch	11.3 ± 3.0	11.3 ± 2.9	= 0.880
Descending aorta	37.3 ± 8.2	38.0 ± 7.9	= 0.400
Thoracic aorta	84.3 ± 16.8	85.6 ± 17.5	= 0.150

**FIGURE 4:**
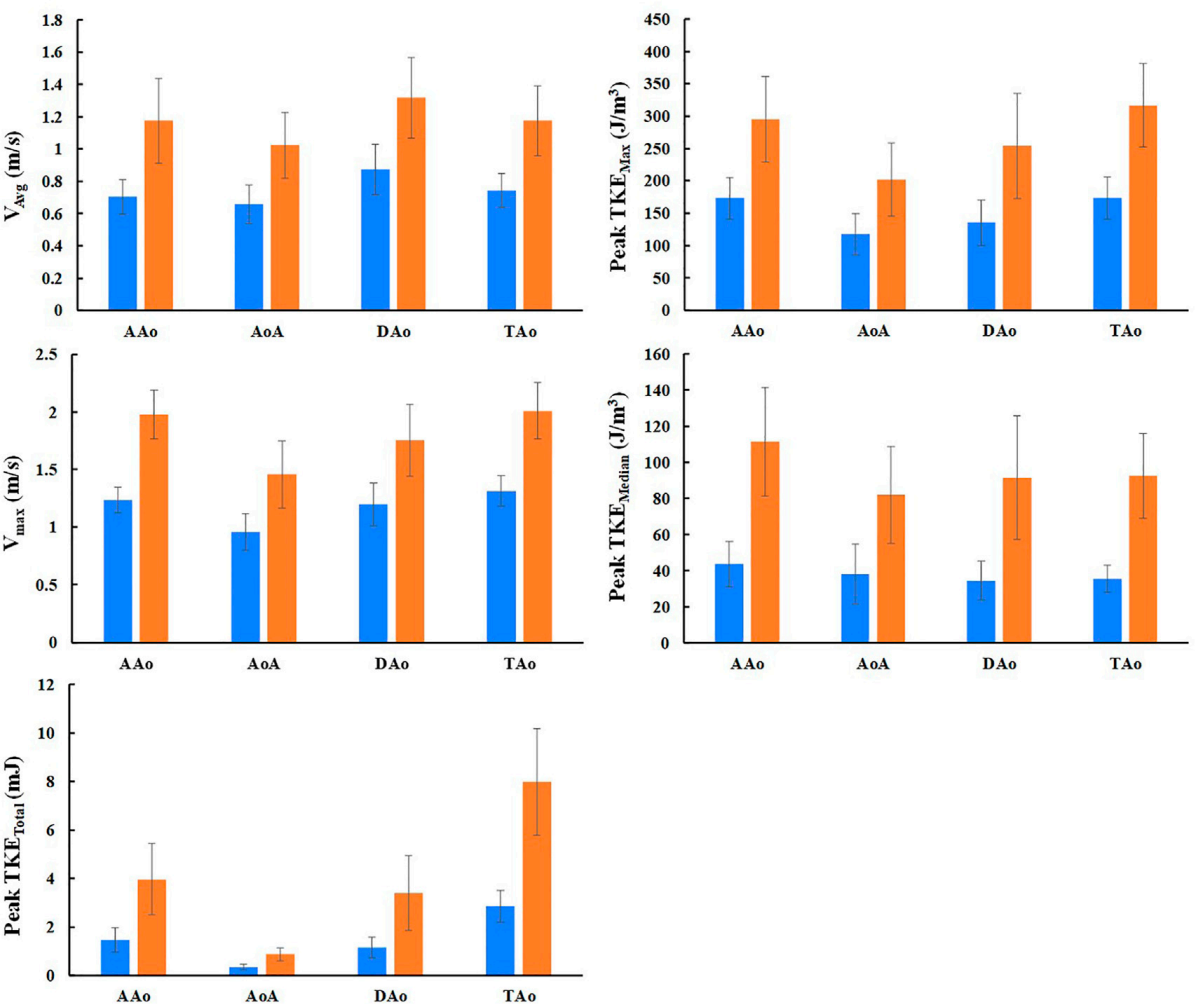
Graphic representation of all hemodynamic parameters, mean and SD. Blue bars are values at rest and orange bars are values with dobutamine infusion; AAo, ascending aorta; AoA, aortic arch; DAo, descending aorta; TAo, thoracic aorta.

Hemodynamic parameters of the aorta for the twelve subjects are shown in Table 3 and Figure 4. With infusion of dobutamine there was a significant increase in all hemodynamic parameters. Peak V_avg_ and peak V_max_ had a 1.5 to 1.6-fold increase with dobutamine stress for AAo and AoA while DAo and TAo had a 1.4 to 1.5-fold increase. Peak TKE_total_ had a 2.8-fold increase in the AAo and TAo, DAo had a 2.9-fold increase while the AoA had a 2.5-fold increase. Peak TKE_max_ had a 1.7 to 1.9-fold and peak TKE_med_ had a 2.2 to 2.7-fold increase.

**TABLE 3:** Hemodynamic parameters for all regions of the thoracic aorta.

	Rest	Dobutamine	*p*-Value
Peak V_avg_ (m/s)			
Ascending aorta	0.70 ± 0.11	1.17 ± 0.26	= 0.002
Aortic arch	0.66 ± 0.12	1.02 ± 0.20	<0.001
Descending aorta	0.87 ± 0.16	1.32 ± 0.25	= 0.002
Thoracic aorta	0.74 ± 0.11	1.17 ± 0.22	<0.001
Peak V_max_ (m/s)			
Ascending aorta	1.24 ± 0.11	1.98 ± 0.21	= 0.002
Aortic arch	0.96 ± 0.16	1.46 ± 0.29	<0.001
Descending aorta	1.20 ± 0.19	1.75 ± 0.31	<0.001
Thoracic aorta	1.31 ± 0.13	2.00 ± 0.25	<0.001
Peak TKE_total_ (mJ)			
Ascending aorta	1.47 ± 0.50	4.09 ± 1.48	<0.001
Aortic arch	0.36 ± 0.12	0.88 ± 0.26	<0.001
Descending aorta	1.16 ± 0.42	3.41 ± 1.54	<0.001
Thoracic aorta	2.86 ± 0.67	8.04 ± 2.20	<0.001
Peak TKEmax (J/m3)			
Ascending aorta	171.4 ± 33.7	316.7 ± 73.4	<0.001
Aortic arch	121.9 ± 43.5	200.9 ± 59.8	= 0.002
Descending aorta	135.3 ± 35.7	253.5 ± 80.6	<0.001
Thoracic aorta	176.3 ± 32.6	334.2 ± 69.0	<0.001
Peak TKE_med_ (J/m3)			
Ascending aorta	43.6 ± 12.6	112.8 ± 30.7	<0.001
Aortic arch	38.1 ± 16.5	82.8 ± 25.8	= 0.002
Descending aorta	34.5 ± 10.9	91.4 ± 34.1	<0.001
Thoracic aorta	35.8 ± 7.2	93.2 ± 23.5	= 0.002

**FIGURE 5:**
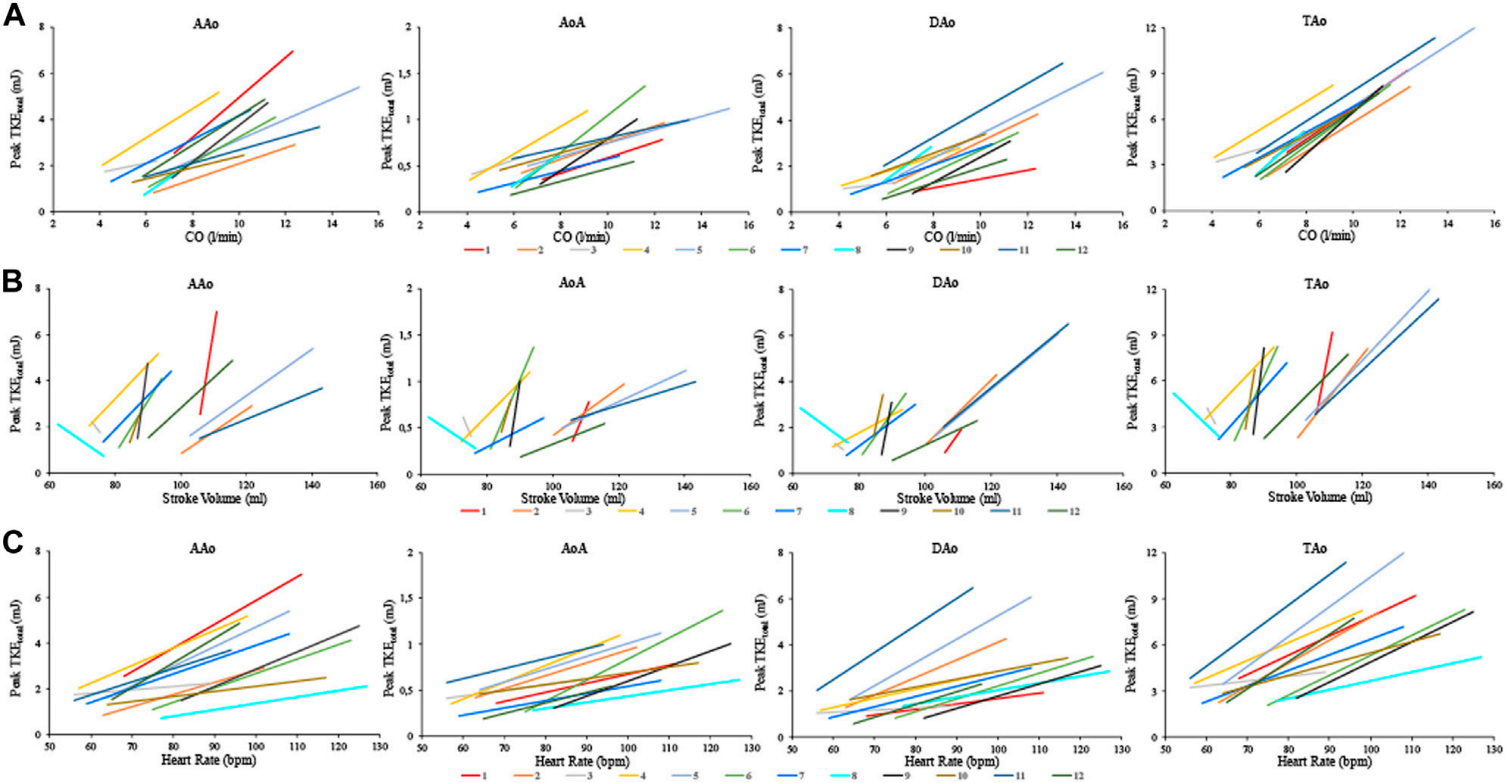
The relation between Peak TKE_total_ and CO **(A)**, left ventricular stroke volume **(B)**, and heart rate **(C)** for each of the 12 healthy subjects in the four aortic regions at rest and with dobutamine infusion. CO, cardiac output; AAo, ascending aorta; AoA, arcus aorta; DAo, descending aorta; TAo, thoracic aorta.

**FIGURE 6:**
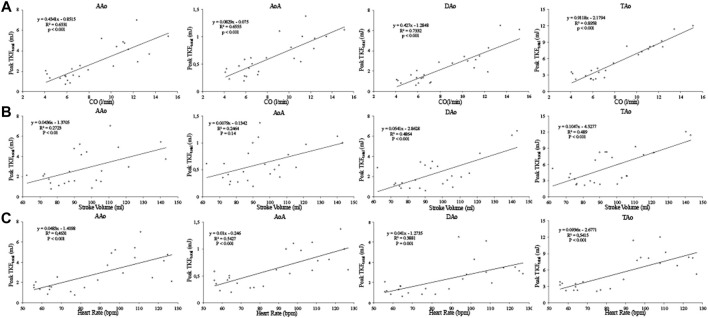
Regression analysis between Peak TKE_total_ and CO **(A)**, left ventricular stroke volume **(B)**, and heart rate **(C)** for the four aortic regions. Values at rest and with dobutamine infusion from each healthy subject are included. CO, cardiac output; AAo, ascending aorta; AoA, aortic arch; DAo, descending aorta; TAo, thoracic aorta (Supplementary Video).

The stress induced change in CO, SV and heart rate and the relation to the change in peak TKE_total_ in each subject is displayed in Figure 5. A linear regression analysis showed that the relation between CO and peak TKE_total_ was significant for all regions (*p* < 0.001), strong for all three separate regions (AAo, *R* ^2^ = 0.65; AoA, *R* ^2^ = 0.66; DAo, *R* ^2^ = 0.73), and very strong for the TAo (*R* ^2^ = 0.90). The relation between SV and peak TKE_total_ was significant for AAo (*p* < 0.01), DAo and TAo (both *p* < 0.001) and non-significant for AoA (*p* = 0.14) with weak relation for AAo and AoA (*R* ^2^ = 0.27 and *R* ^2^ = 0.25, respectively) and moderate relation for DAo and TAo (*R* ^2^ = 0.49). The relation between heart rate and peak TKE_total_ was significant (*p* < 0.001) and moderate for all regions (AAo, *R* ^2^ = 0.46; AoA, *R* ^2^ = 0.54; DAo, *R* ^2^ = 0.39; TAo, *R* ^2^ = 0.54). (Figure 6). The relation between blood pressure and peak TKE_total_ was significant for AAo, AoA (*p* < 0.01), DAo and TAo (*p* < 0.001) with weak correlation for AAo and AoA (*R* ^2^ = 0.33 and *R* ^2^ = 0.36, respectively), strong correlation for DAo (*R* ^2^ = 0.67) and moderate correlation for TAo (*R* ^2^ = 0.59).

Differences between aortic regions in the hemodynamic parameters are shown in Table 4 and Figure 4. Overall, there were slightly more inter-region differences at rest than at stress. At rest; peak V_avg_ was higher in DAo vs. AoA and AAo, peak V_max_ was lower in AoA compared to the three other regions, peak TKE_max_ were higher in TAo and AAo vs. AoA and DAo. With dobutamine stress; peak V_avg_ was lower in AoA vs. DAo, peak V_max_ and peak TKE_max_ were lower in AoA vs. AAo and TAo.

**TABLE 4:** Parameter difference between aortic regions.

	Rest	Dobutamine
**Peak V_avg_ (m/s)**		
Ascending aorta	0.70 ± 0.11°°	1.17 ± 0.26
Aortic arch	0.66 ± 0.12°°°	1.02 ± 0.20°°
Descending aorta	0.87 ± 0.16	1.32 ± 0.25
Thoracic aorta	0.74 ± 0.11	1.17 ± 0.22
**Peak V_max_ (m/s)**		
Ascending aorta	1.24 ± 0.11^•••^	1.98 ± 0.21^•••^
Aortic arch	0.96 ± 0.16°°***	1.46 ± 0.29°°***
Descending aorta	1.20 ± 0.19	1.75 ± 0.31
Thoracic aorta	1.31 ± 0.13	2.00 ± 0.25
**Peak TKE_total_ (mJ)**		
Ascending aorta	1.47 ± 0.50^•••^ ***	4.09 ± 1.48^•••^ ***
Arcus aorta	0.36 ± 0.12°°°***	0.88 ± 0.26°°***
Descending aorta	1.16 ± 0.42***	3.41 ± 1.54***
Thoracic aorta	2.86 ± 0.67	7.98 ± 2.20
**Peak TKE_max_ (J/m3)**		
Ascending aorta	171.4 ± 33.7^•••^°	316.7 ± 73.4^••^
Aortic arch	121.9 ± 43.5***	200.9 ± 59.8***
Descending aorta	135.3 ± 35.7*	253.5 ± 80.6*
Thoracic aorta	176.3 ± 32.6	334.2 ± 69.0
**Peak TKE_med_ (J/m3)**		
Ascending aorta	43.6 ± 12.6	112.8 ± 30.7
Aortic arch	38.1 ± 16.5	82.8 ± 25.8
Descending aorta	34.5 ± 10.9	91.4 ± 34.1
Thoracic aorta	35.8 ± 7.2	93.2 ± 23.5

Results of post-hoc analysis between aortic regions for hemodynamic parameters. Significant difference between aortic regions is indicated by: *p < 0.05 vs. TAo, **p < 0.01 vs. TAo, ***p < 0.001 vs. TAo, ^•^ *p* < 0.05 vs. AoA, ^••^ *p* < 0.01 vs. AoA, ^•••^ *p* < 0.001 vs. AoA, **°** *p* < 0.05 vs. DAo, *p* < 0.01 vs. DAo, *p* < 0.001 vs. DAo.

Intraobserver variability for the velocity and TKE-parameters were excellent (ICC = 0.96–1.00). Interobserver variability for these parameters were also excellent (ICC = 0.91–1.00).

## Discussion

Healthy subjects underwent 4D Flow MRI scans at rest and with dobutamine stress to investigate the impact of heart rate and inotropic state on hemodynamic parameters in the thoracic aorta. With cardiac stress, an increase of flow velocity and turbulence intensity were observed in all regions of the thoracic aorta.

The findings of this experimental study demonstrate that TKE increased with a similar factor in all aortic regions in this cohort of healthy individuals. Both heart rate and LV stroke volume increased with cardiac stress and related weakly to moderately to TKE for the different aortic regions and, further, heart rate overall showed a slightly stronger relation to TKE compared to LV stroke volume. However, the relation between the combination of heart rate and stroke volume (i.e., cardiac output) and TKE was strong for the three separate aortic regions and very strong for the whole thoracic aorta. Dobutamine infusion enhance both chronotropy and inotropy, but as demonstrated by Ahonen et al. (2008) its effect on stroke volume is achieved at low concentrations, and thus, it seems reasonable that stroke volume does not show a stronger relation to turbulence intensity than heart rate in this setting where the peak dobutamine dose was 15–30 μg/kg/min. The very strong relation between CO and turbulence intensity is interesting, especially with the rather heterogenous characteristics of the current study cohort of healthy volunteers.

In comparison to previous studies, the TKE_total_ values at rest in our healthy subjects appear to be lower, a discrepancy that may in part be explained by differences in gender and age between the populations. The definition of AAo differs slightly between studies, and if AAo and AoA in our work are combined, the resulting region is comparable with the AAo of other works. In our study, including eight females and four males with the mean age of 33 years, peak TKE_total_ was 1.8 mJ in this combined region. Ha et al. (2018) studied two groups of men with different ages (mean age 24, n = 22; mean age 71, n = 20) and showed that TKE_total_ was 3.7 mJ in the younger group and 6.4 mJ the older group. Moreover, Binter et al. (2017) investigated peak TKE_total_ in ten healthy subjects, five females and five males, with a mean age of 69 years, and found values of 4.8 mJ for a region slightly larger than the AAo and AoA combined. We demonstrate a strong relation between peak TKE_total_ and CO, and as female in general have smaller CO than males, this may contribute to the lower peak TKE_total_ in our study, which includes a majority of female subjects. Ha et al. also showed that older men have higher TKE in the AAo than younger men, which could in part explain the higher values observed by Binter et al. in addition to the gender distribution. Furthermore, the methods of segmentation could also impact the results, for example, we excluded the branches in the aortic arch and branching in arteries may promote turbulence.

In previous works, peak TKE_med_ has not been reported as frequently as other TKE parameters. However, it is a promising parameter for the assessment of the turbulence intensity in a region as it allows for comparison between different regions and individuals (Ha et al., 2018). This is in contrast to peak TKE_total_ which measures the total TKE in a region and can thus be expected to increase with larger anatomic dimensions. The peak TKE_med_ values in this study are similar to what Ha et al. found, especially compared to the younger cohort, implying that the difference observed in peak TKE_total_ between the studies can in part be volume related.

Although TKE increases with a similar factor in all aortic regions the results also show inter-region differences. Overall, the AoA showed the lowest velocities and TKE values, and these findings could be related to the curvature of this region and blood flow reduction due to flow to the supra-aortic arteries. The region with the highest turbulence intensity differs between subjects where some have the highest TKE in the AAo and some in the DAo. These inter-region differences in the distribution of the highest TKE values could be attributed to characteristics of the subjects such as age. Ha et al. presented in their study that older men were prone to have higher peak TKE_med_ in the AAo and lower TKE_med_ distal to the AAo, while the younger had similar levels of peak TKE_med_ in the AAo as the older, but TKE_med_ was maintained throughout the rest of the aorta (Ha et al., 2018). The current results suggest a similar trend with distribution of higher TKE_med_ in the AAo vs. the DAo in the three oldest subjects (43–58 years). The younger subjects in our study had maintained or even higher peak TKE_med_ values in the DAo compared to the AAo. However, the current study cohort is too small to allow any conclusions of the relation between age and hemodynamic parameters.

Peak TKE_max_ in the AAo at rest was 171 J/m^3^ in this study which is similar to 100–230 J/m^3^ shown in previous studies in healthy subjects (Dyverfeldt et al., 2008; Binter et al., 2013; Ha et al., 2018). With dobutamine stress the value increased to 317 J/m^3^ for AAo, but this is still lower than values of 450–1089 J/m^3^ found at rest in patients with aortic valve stenosis, aortic coarctation and aortic valve implant (Dyverfeldt et al., 2008; Arzani et al., 2012; Binter et al., 2013; Dyverfeldt et al., 2013).

In the work of Binter et al. (2017), they analyzed and reported TKE values of both peak systole and values integrated over systole in the aortic root and arch. In the current work, we decided to focus on peak TKE so as to study the temporally maximal total TKE in the whole thoracic aorta, as the main goal of the study was to investigate how heart rate and inotropy effects turbulence. It is reasonable to believe that the current peak TKE is related to the TKE parameter integrated over systole. In the same work, Binter et al. normalized TKE to stroke volume. This index (or TKE normalized to CO) may be useful for investigation of additional factors that are linked to the development of TKE and provide some aspects of the efficiency of flow in the circulatory system.

Both TKE and viscous energy loss can be calculated from a 4D flow MRI derived velocity field. TKE is the mean kinetic energy associated with turbulent fluctuations in the flow and is expressed in Joule or Joule/m^3^, whereas viscous energy loss is the kinetic energy converted to thermal energy and is expressed in Joule/s or Watt. Both kinetic energy and TKE are in principle converted to thermal energy, but due to limited spatiotemporal resolution the computation of viscous energy loss from 4D flow MRI does not include the dissipation of TKE (Barker et al., 2014). Quantification of dissipation of TKE, which dominates in turbulent flow, requires higher temporal and spatial resolution or the measurement of turbulent shear stresses (Ha et al., 2016b).

Dobutamine is a widely used pharmacological stress agent with inotropic and chronotropic effects on the heart. In the cardiac MRI setting, motion of the thorax (i.e., the heart) during data acquisition is challenging for obtaining data with good quality. Dobutamine stress leads to motion of the thorax but to a lesser degree compared to physical exercise. However, pharmacological stress does not elicit a natural stress response on the cardiovascular system as compared to physical stress. The 60% increase of heart rate as the endpoint of dobutamine infusion dose is a trade-off between eliciting sufficient hemodynamic effects and performing a cardiac MRI scan that provide data with sufficient quality. For instance, higher dobutamine dose leads to more respiratory motion that has to be corrected for, higher demands on temporal resolution, and increasing discomfort for the subject.

This experimental study was an attempt to provide novel physiological aspects on how TKE parameters are impacted by chronotropy and inotropy. With further development of data acquisition and post processing, several TKE parameters have the potential to be used to assess cardiovascular pathophysiology and add to patient management. Several cardiovascular diseases can cause turbulent blood flow in the thoracic aorta (Dyverfeldt et al., 2008; Arzani et al., 2012; Binter et al., 2013; Dyverfeldt et al., 2013; Lantz et al., 2013; Ha et al., 2016a; Binter et al., 2017; Habash and Vallurupalli 2017). Turbulent flow in the thoracic aorta has also been associated with development of pathological processes such as aneurysms, dissections and atherosclerosis (Sutera 1977; Davies et al., 1986; Davies 1995; Becker et al., 2001; Ekaterinaris et al., 2006; Chiu and Chien 2011; Hollestelle et al., 2011; Numata et al., 2016). Assessment of TKE can potentially be of added value in the evaluation of cardiovascular stenosis and valvular regurgitation, and assessment of risk for damage to surrounding vascular tissue and blood constituents. These can currently not sufficiently be assessed using conventional methods, and do therefore have to rely on future clinical studies.

Furthermore, TKE quantification during dobutamine stress could add to the detection of abnormalities which are subtle at rest and potentially aid in the decision to initiate preventive management.

### Limitations

This study cohort is relatively small, however, the study has an experimental design and utilizes state-of-the-art MRI techniques. Moreover, the participants had a wide range of age and a mix of gender and can be considered as a pilot study of the research field. Also, for accurate quality of 4D flow data, the subject needs to remain still during data acquisition and any movement of the heart must be compensated for in the post processing. We have used established registration techniques to account for this issue, but with data acquisition during dobutamine stress the challenges are more pronounced.

We used a VENC of 140 cm/s to be able to assess hemodynamic parameters including velocity. For measurements of the moderately elevated aortic TKE values in the present study, a VENC of 140 cm/s is high, but acceptable. Noise affects TKE measurements in two ways. For SNR>3, noise in MR magnitude images is Gaussian distributed and leads to uncertainties in TKE, similar to how noise leads to uncertainties in PC-MRI velocity measurements. For SNR<3, noise in MR magnitude images approaches a Rayleigh distribution, which manifests as a so-called noise floor. The noise floor limits the maximum TKE that can be resolved (Dyverfeldt et al., 2009). Setting the VENC in TKE measurements to a relatively high value, as done in this study, minimizes the risk of TKE underestimation due to the noise floor effect. At the time of data acquisition, we did not have access to a dual- or multi-VENC sequence. However, such a sequence would have prolonged scan time and, as dobutamine can be very challenging even for healthy volunteers, we sought to use a relatively short scan protocol.

## Conclusion

This study shows how 4D flow based hemodynamic parameters, and especially TKE, in the healthy thoracic aorta is affected by dobutamine stress. Increased chronotropy and inotropy increases TKE with a similar factor in all aortic regions, and the level of TKE is strongly related to cardiac output. Assessment of TKE with cardiac stress may serve as a marker of risk for developing aortic disorders before they are clinically manifested.

## Data Availability

The raw data supporting the conclusions of this article can be made available by the authors, upon reasonable request. Requests to access the datasets should be directed to the corresponding author.

## Supplementary Material

Supplementary Video S1 Visualization of TKE in the thoracic aorta at rest (left) and dobutamine stress (right) in a 20 year old healthy female.

Supplementary Video S2 Visualization of TKE in the thoracic aorta at rest (left) and dobutamine stress in a 56 year old healthy female.
